# Emerging Themes in the Molecular Pathogenesis of Enterotoxigenic *Escherichia coli*

**DOI:** 10.1093/infdis/jiab359

**Published:** 2021-07-17

**Authors:** James M Fleckenstein, Alaullah Sheikh

**Affiliations:** 1 Department of Medicine, Division of Infectious Diseases, Washington University in St Louis, School of Medicine, St Louis, Missouri, USA; 2 Infectious Disease Section, Medicine Service, St Louis Veterans Affairs Health Care System, St Louis, Missouri, USA

**Keywords:** *Escherichia coli*, enterotoxigenic, malnutrition, child, brush border, microvilli, bacterial vaccines, enterotoxins, cyclic AMP, CEACAM6, Mucin 2

## Abstract

Enterotoxigenic *Escherichia coli* (ETEC) are ubiquitous diarrheal pathogens that thrive in areas lacking basic human needs of clean water and sanitation. These genetically plastic organisms cause tremendous morbidity among disadvantaged young children, in the form of both acute diarrheal illness and sequelae of malnutrition and growth impairment. The recent discovery of additional plasmid-encoded virulence factors and elucidation of their critical role in the molecular pathogenesis of ETEC may inform new approaches to the development of broadly protective vaccines. Although the pathogens have been closely linked epidemiologically with nondiarrheal sequelae, these conditions remain very poorly understood. Similarly, while canonical effects of ETEC toxins on cellular signaling promoting diarrhea are clear, emerging data suggest that these toxins may also drive changes in intestinal architecture and associated sequelae. Elucidation of molecular events underlying these changes could inform optimal approaches to vaccines that prevent acute diarrhea and ETEC-associated sequelae.

The enterotoxigenic *Escherichia coli* are common pathogens that comprise a genetically diverse diarrheagenic *E. coli* pathovar defined by the production of heat-labile toxin (LT) and/or heat-stable toxin (ST). It is estimated that worldwide hundreds of millions of symptomatic enterotoxigenic *E. coli* (ETEC) infections occur annually [1], a burden predominantly borne by young children of low-middle-income countries. Here, ETEC remain a major cause of deaths due to acute diarrheal illness. Abundant in low-middle-income regions where clean water and sanitation remain limited, ETEC are perennially the most common pathogen isolated from travelers with infectious diarrhea. Surprisingly, however, they have also been associated with outbreaks [2, 3] and sporadic cases in more developed areas, including the United States, where recently introduced culture-independent methods may accelerate their identification [4, 5].

## CANONICAL VIEW OF ETEC VIRULENCE

These pathogens were originally identified now >5 decades ago in patients presenting with severe choleralike diarrheal syndromes [6]. Early investigation in these patients with non-*Vibrio* cholera in India and Bangladesh soon revealed the presence of *E. coli* that produced a heat-labile toxin (LT) LT similar to cholera toxin, and subsequently the small peptide STs, as well as the first of many plasmid-encoded fimbrial colonization factors (CFs). A canonical view of ETEC virulence soon evolved, in which these pathogens simply adhere to intestine via CFs to deliver their toxin payloads, and much of the ensuing investigation of ETEC related to discovery and characterization of additional plasmid-encoded CFs or coli surface (CS) antigens. To date, >25 antigenically distinct CF/CS antigens have been defined. Elucidation of the structural biology and biogenesis of these antigens suggests that development of a broadly protective vaccine would require a multivalent approach incorporating multiple adhesins and toxoid molecules [7]. Approaches to vaccine development have focused primarily on these antigens and LT. Nevertheless, data generated over the past few years have highlighted major gaps in our understanding of these extraordinarily common pathogens to demonstrate that ETEC are far more complex than had been appreciated.

## ESSENTIAL ELEMENTS OF ETEC AS A SUCCESSFUL PATHOGEN

In effect, ETEC molecular pathogenesis can be viewed as the summary of events that enable these bacteria to reach the small intestine, traffic to the mucosal surface, engage epithelial cell receptors and ultimately deliver their LT and/or ST payloads. This process ultimately requires a coordinated division of labor between highly conserved core elements encoded the *E. coli* chromosome and the ETEC pathovar-specific features encoded on virulence plasmids [8].

## IMPORTANCE OF CHROMOSOMALLY ENCODED VIRULENCE TRAITS

With time, it has become clear that ETEC rely on a number of chromosomally encoded features. Virtually all ETEC are motile and can be serotyped according to their flagellar H antigens, and flagellar motility is essential to successful delivery of both LT and ST. The flagellar assembly apparatus is encoded on the chromosome, as are genes encoding type 1 pili common to all *E. coli* [9], and those encoding additional outer membrane surface adhesins [10]. Likewise, although the toxins that define ETEC are plasmid encoded, the type II secretion system for LT [11], and the type I TolC secretion system responsible for export of ST [12] are both encoded on the chromosome. Remarkably, before the discovery of its cognate export system, ETEC were thought to simply release LT by lysis, as expression of the structurally similar cholera toxin in a laboratory strain of *E. coli* (which happened to lack the type II secretion system) failed to export the toxin. Fortunately, we now know that these chromosomally encoded features work in a highly orchestrated and cooperative fashion [8] with plasmid-borne pathovar-specific virulence traits to facilitate toxin delivery (Table 1).

**Table 1.** Enterotoxigenic *Escherichia coli* Virulence Factors

Virulence Factor/Locus	Gene Location	Description and Known Dominant Functions	ETEC Specific?
Canonical ETEC virulence traits			
CFs/CS antigens	Plasmid	Fimbrial and afimbrial structures that promote adhesion and small-intestinal colonization	Yes
LT	Plasmid	ADP ribosylating toxin, activates cAMP production to alter ion channels	Yes
STs (STh and STp)	Plasmid	Binds guanylate cyclase C to activate cGMP production to alter ion channels	Yes
Chromosomally encoded *Escherichia coli* virulence traits			
Flagella	Chromosome	Motility; essential for toxin delivery	No
Type 1 pili	Chromosome	Adhesion to mannosylated glycoproteins	No
EaeH	Chromosome	Outer membrane protein/adhesin	No
YghJ	Chromosome	Metalloprotease	No
T2SS	Chromosome	Responsible for secretion of LT and YghJ	No
TolC T1SS	Chromosome	Responsible for secretion of STh and STp	No
Noncanonical ETEC virulence factors			
EatA	Plasmid	Mucin-degrading serine protease autotransporter protein	Yes^a^
EtpB, EtpA, EtpC	Plasmid	2-Partner secretion system responsible for export of EtpA, an extracellular adhesin that binds to GalNAc and blood group A glycans	Yes

Abbreviations: ADP, adenosine diphosphate; cAMP, cyclic adenosine monophosphate; CFs, colonization factors; cGMP, cyclic guanosine monophosphate; CS, coli surface; EatA, ETEC autotransporter A; ETEC, enterotoxigenic *Escherichia coli*; GalNAc, N-acetylgalactosamine; LT, heat-labile toxin; STs, heat-stable toxins; T1SS, type I secretion system; T2SS type II secretion system.

^a^Also found in some *Shigella* spp., including *Shigella sonnei*.

## IDENTIFYING AND CHARACTERIZING ADDITIONAL PLASMID-ENCODED ETEC VIRULENCE FACTORS

**Figure 1.**
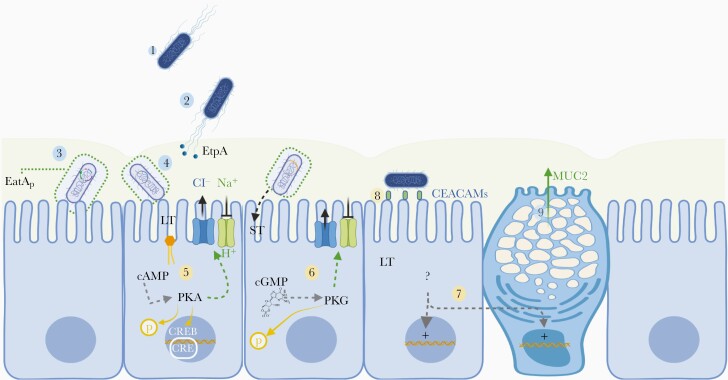
Summary of steps in the molecular pathogenesis of enterotoxigenic *Escherichia coli* (ETEC). Step 1: ETEC are propelled to the small-intestinal lumen via peritrichous flagella. Step 2: ETEC engage the mucin overlying small-intestinal epithelia cells, in part mediated by EtpA bridging of the bacterial flagella with glycans present in mucin. Step 3: Degradation of MUC2 by the proteolytically active EatA passenger domain (EatA_p_) permits bacterial access to the epithelial surface. Steps 4 and 5: ETEC engage enterocytes via plasmid-encoded colonization factor molecules in addition to EtpA, where delivery of heat-labile toxin (LT) activates production of cellular cyclic adenosine monophosphate (cAMP), which in turn activates protein kinase A (PKA). PKA catalytic subunits phosphorylates sodium (Na^+^) and chloride (Cl^−^) channels, resulting in the net export of salt and water into the intestinal lumen and diarrhea. PKA also phosphorylates other cellular target proteins, and enters the nucleus to alter transcription via cAMP response element (CRE) binding protein (CREB). Step 6: Heat-stable toxin (ST) binds to guanylate cyclase C to elicit the production of cyclic guanosine monophosphate (cGMP), activating protein kinase G (PKG), which phosphorylates ion channel proteins and other targets. Steps 7–9: LT modulates the transcription of multiple genes including those encoding CEACAMs, which that then serve as receptors for ETEC expressing type 1 fimbriae and MUC2 enhancing the mucin barrier. Abbreviation: H^+^, . (Figure created with BioRender.com.)

The identification of additional surface-expressed antigens has expanded the repertoire of ETEC virulence factors and contributed to our understanding of ETEC molecular pathogenesis. Two plasmid-encoded loci, discovered in a search for novel secreted molecules, have emerged as important virulence factors. These include EatA, a member of the serine protease autotransporter of the Enterobactericiae family [13], and the *etpBAC* 2-partner secretion locus responsible for export of EtpA, an extracellular adhesin [14]. (Figure 1)

Although the layer of secreted mucin in the small intestine is relatively thin compared with that in the colon, it is likely that this effectively prevents effective engagement by pathogenic bacteria including ETEC, and that LT, like cholera toxin, can induce the production and release of mucin from goblet cells. Studies have shown that the secreted passenger domain of EatA, which contains both mucin-binding activity as well as the serine protease catalytic triad, degrades MUC2, the major mucin secreted by intestinal goblet cells.

MUC2 is a very large glycoprotein that has been dimerized at its C-terminal end and is thought to form complex trimeric structures at the N-terminus of the molecule. This results in hexameric arrays of MUC2 arranged in sheets [15] that in effect trap the bacteria and prevent pathogen-host engagement. Data emerging from EatA-mediated degradation of MUC2 suggest that this conserved protease, as well as SepA, a homologous protein from *Shigella flexneri*, work by cleaving MUC2 to effectively dissolve the protective sheets of mucin. ETEC equipped with EatA can therefore overcome the mucin barrier, allowing these pathogens to engage in productive pathogen-host interactions essential for toxin delivery [16].

EtpA promotes these interactions by acting as a molecular bridge [17] between the bacteria and host cell glycans present on mucins [18] and intestinal epithelial cells. Like a host of bacterial adhesins, including another 2-partner secretion protein, filamentous hemagglutinin of *Bordetella pertussis,* part of acellular pertussis vaccines, EtpA also possesses hemagglutinating activity. In the case of EtpA, this activity is very specific for human A blood group erythrocytes. Because blood group A glycans are also present on intestinal epithelia, the preferential binding of EtpA may explain observations of more severe illness observed among those in blood group A+, including naturally infected young children [19] and human volunteers challenged with ETEC [20].

Several findings attest to the importance of these more recently discovered “noncanonical” virulence proteins in the molecular pathogenesis of ETEC. First, both antigens discovered in ETEC H10407 [13, 14], an early isolate from a patient with severe choleralike diarrhea, have been identified in multiple isolates from severe diarrheal illness, and they are conserved and have been retained over time among a large and geographically dispersed population of ETEC [21–24]. Both antigens are highly immunogenic in human volunteers challenged with H10407 [25, 26] and naturally infected hosts [24] suggesting that they are expressed in vivo. Notably, recent analysis of strains obtained from a birth cohort of young children in Bangladesh indicate that both molecules are significantly associated with symptomatic infection [27]. Conversely, antibodies against either antigen were associated with asymptomatic colonization. Collectively, the data emerging from molecular pathogenesis as well as clinical studies seem to support a role for these recently discovered “noncanonical” antigens in virulence.

## POTENTIAL IMPORTANCE OF NONCANONICAL ANTIGENS AS VACCINE TARGETS

These recent studies of ETEC molecular pathogenesis may afford new approaches to the development of a broadly protective vaccine. The conservation of these more recently discovered highly immunogenic molecules suggests that they may provide alternative targets for vaccine development. Recently, available ETEC genomes were mined to identify potential surface antigens that were conserved in at least 40% of isolates, and these were then spotted onto protein microarrays to identify those recognized during the course of infection [27]. Interestingly, and somewhat surprisingly, these “open-aperture” immunoproteome studies demonstrated relatively few immunogenic pathovar-specific proteins, among them EtpA and EatA. Taken together, these data suggest that targeting these 2 molecules could help to overcome some of the hurdles inherent in targeting the less highly conserved CF antigens. Molecular epidemiology of >1000 strains from disparate sources indicate that a vaccine targeting just 2 antigens, CS6 and EtpA would likely encompass more than three-quarters of all ETEC [21].

Studies are also underway to elucidate the molecular structure of EtpA by cryoelectron microscopy, map the antibody responses onto the antigen, and determine the functional activity of monoclonal antibodies which recognize specific epitopes. These collaborative “structural vaccinology” efforts should facilitate the identification of critical regions of this large (approximately 170-kD) molecule to target in a polyvalent vaccine.

## UTILITY OF INTESTINAL ORGANOIDS AS A NOVEL MODEL IN VITRO SYSTEM FOR ETEC PATHOGENESIS

**Figure 2.**
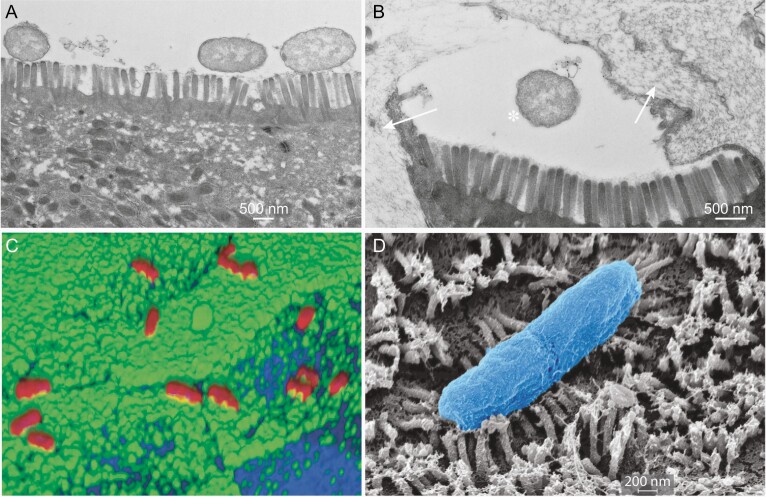
*A,* Transmission electron microscopic image of enterotoxigenic *Escherichia coli* (ETEC) adhering to the apical surface of small-intestinal enteroids derived from human ileum. *B,* ETEC (*asterisk*) is shown in cross-section, approaching an enterocyte flanked by 2 goblet cells that have released mucin (*arrows*). *C,* ETEC adhering to the apical surface of small-intestinal enteroids in regions of CEACAM6 expression. *D,* ETEC (H10407 strain; isolated from a patient with choleralike diarrhea in Bangladesh) adhering to the brush border formed by microvilli on the surface of small-intestinal enteroids propagated from a human (blood group A+) small intestine (scanning electron microscopy; original magnification ×27 700).

Seminal studies by Hans Clevers and his laboratory in the Netherlands defined the signals required to maintain and propagate intestinal epithelial cells from stem cells [28–30]. Stem cells obtained from biopsies performed during routine endoscopic procedures can now be preserved, expanded, and differentiated to recapitulate many of the features of normal human intestinal epithelia normally encountered by the bacteria. Unlike transformed cells derived from gastrointestinal malignant neoplasms, which have been widely used in the past to study ETEC pathogen-host interactions, intestinal organoids reflect the population of cells normally present in the intestinal epithelium, including Paneth cells, goblet cells, enteroendocrine cells, and, importantly, enterocytes with a well-defined brush border and glycocalyx (Figure 2A). Importantly, with access to large biobanks of cells from multiple individuals, rigorous data can now be generated and the importance of features unique to the original host can be examined in detail.

These small-intestinal “enteroid” systems have already proved to be extraordinarily useful in exploring pathogen-host interactions. Their ability to recapitulate important features of human biology is illustrated in experiments showing that ETEC deliver toxins more efficiently to blood group A enterocytes, reflecting increased disease severity in human volunteers and naturally infected children who express blood group A antigens [20].

In addition, goblet cells present in the enteroids produce significant amounts of MUC2 mucin, which normally serves as a protective barrier to mitigate interaction of ETEC with enterocytes goblet cells (Figure 2B). Data emerging from experiments with small-intestinal enteroids as well as with surgical explants of human small intestine demonstrate that EatA plays a critical role, by eliminating this protective barrier to permit access of the bacteria to the surface of enterocytes, where they can adhere and deliver their toxin payloads.

## ENIGMATIC SEQUELAE ASSOCIATED WITH ETEC INFECTION

While the mortality rate associated with acute diarrheal diseases has declined appreciably since the introduction of oral rehydration therapy and other measures, even less severe episodes of ETEC diarrhea can exert lasting effects on children, including growth faltering, and malnutrition [31]. Although ETEC, as well as other pathogens, have been repeatedly linked to these sequelae in children, the molecular events underlying these nondiarrheal conditions collectively referred to as *environmental enteric dysfunction* (EED) or *environmental enteropathy,* remain very poorly understood.

Interestingly, another enigmatic condition linked to toxin-producing *E. coli,* tropical sprue, remains the most common cause of chronic small-intestinal malabsorption and diarrhea in areas of high ETEC prevalence including India [32]. Notably, multiple studies in the 1970s identified “toxin producing *E. coli*” in luminal aspirates from patients with tropical sprue [33]. Unfortunately, these studies relied on rabbit ileal loops to demonstrate enterotoxin production, and we lack definitive molecular characterization of isolates from these patients.

Both EED and tropical sprue are characterized by shortening of the small-intestinal villi, lengthened crypts, mucosal inflammation, and nutrient malabsorption [34]. Despite these common features and epidemiologic associations connecting these nondiarrheal sequelae to ETEC, we lack a molecular understanding of how these pathogens might promote the development of intestinal changes characteristic of enteropathy, or whether ETEC alone are sufficient to elicit them in addition to causing diarrhea. Essentially, we have yet to fulfill “molecular Koch’s postulates”[35] that firmly establish ETEC as the causative agents.

## AN EXPANDED ROLE FOR TOXINS

Notably, both ETEC enterotoxins target intestinal epithelia to activate the production of cyclic nucleotides that serve as major cellular “second messengers” involved in the modulation of multiple cellular pathways. Soon after the discovery of LT, it was recognized that it stimulates the production of cyclic adenosine monophosphate (cAMP) by adenylate cyclase [36]. Intracellular accumulation of cAMP leads to activation of the protein kinase A heterotetramer by liberating its 2 catalytic subunits from regulatory subunits. The catalytic protein kinase A subunits are then free to phosphorylate cellular targets, including chloride and sodium ion channels, to modulate their activity, resulting in the net release of salt and water into the lumen of the small intestine. In addition, the free catalytic subunits also move into the nucleus. where they phosphorylate and activate the cAMP response element binding protein (CREB) transcription factor. Binding of CREB to CRE sites in promotor regions can potentially modulate the activity of thousands of genes [37].

Similarly, ST, binds to guanylate cyclase C on the surface of intestinal epithelial cells, where it activates production of cyclic guanosine monophosphate, to activate protein kinase G, which in turn can phosphorylate multiple cellular proteins in addition to those directly related to modulation of ion channels and development of diarrhea. Remarkably, however, despite the capacity for these toxins to induce pleiotropic effects on the host cell via their impact on critical “second messenger” [38], pathways, we presently have very little information regarding other potential effects that LT- and ST-induced signaling may have on the host, other than diarrhea.

Interestingly, LT was previously shown to play an important role in promoting ETEC adhesion to intestinal epithelial cells in vitro [39] and small-intestinal colonization in animal models [40, 41]. More recent investigations demonstrate that intracellular increases in cAMP lead to enhanced production of a number of glycoproteins known as Carcinoembryonic Cell Adhesion Molecules (CEACAMs), expressed on the surface of small-intestinal epithelial cells [42]. These proteins, in particular CEACAM6 (Figure 2C), then serve as receptors for FimH, the tip adhesin molecule expressed at the ends of chromosomally encoded type 1 pili [9]. In effect, ETEC use the plasmid-encoded LT to change the surface architecture of target host enterocytes, enhancing production of their own receptor.

While these events *transiently* benefit the pathogen, these architectural changes may come at the expense of the host, as they are occurring on the surface of enterocytes, the major site of nutrient absorption in the human small intestine. Enterocytes are propagated from stem cells in the small-intestinal crypts, and they migrate to the distal end of small-intestinal villi as they mature, eventually being released from the extracellular matrix and shed from the villus tip to undergo programmed cell death, or *anoikis* [43], a process inhibited by deregulated CEACAM6 expression [44]. Alteration of this normal homeostatic mechanism of intestinal villi could ultimately prove to be detrimental to the host. Conversely, enhanced expression of CEACAMs by the epithelia could also prove to be important as a host defense strategy, as large amounts of CEACAMs shed in stool [45] could serve as molecular decoys that mitigate binding of ETEC to intestinal epithelia.

## ETEC TOXINS AS POSSIBLE DRIVERS OF ENTEROPATHY

At a microscopic level, both tropical sprue and EED are characterized morphologically by substantial alterations in the small-intestinal villous architecture, characterized by blunting of the villi. The epithelial surface of intestinal villi is comprised of enterocytes, goblet cells, tuft cells, and enteroendocrine cells that differentiate as they propagate up the villus surface from progenitor cells in the crypts of Lieberkuhn, where intestinal stem cells and Paneth cells reside. Enterocytes are the most abundant cells in intestinal epithelia, and the major site of nutrient absorption. At an ultrastructural level, the intestinal “brush border” is normally formed by densely packed regular arrays of microvilli at the surface of enterocytes. In essence, the microvilli are extensions of the enterocyte apical plasma membrane surrounding a central core of actin microfilaments. These protrusions greatly increase the available surface area at luminal surface of the intestine, allowing for maximal nutrient absorption. As such, insults that interfere with the development of microvilli can potentially result in malnutrition.

Intriguingly, transmission electron microscopy of small-intestinal biopsy specimens from patients with tropical sprue, obtained by Mathan et al in the 1970s [46], demonstrated marked derangement of the enterocyte brush border with irregular, short, and sparse microvilli. Notably, this group found that in patients with cholera [47], the ultrastructural derangements of the small-intestinal epithelia were remarkably similar to those with tropical sprue, raising the possibility that these changes are toxin mediated.

Not surprisingly, given the importance of cAMP as a second messenger in the cell, we found by RNA sequencing that treatment of small-intestinal enteroids with LT, a close homologue of cholera toxin, results in the modulation of many cellular genes. Among the hundreds of genes altered by LT were those encoding proteins needed for the biogenesis of microvilli [48], and transmission electron micrographs of enterocytes look astoundingly like those seen in tropical sprue and cholera. Moreover, many of the transporters for vitamins and nutrients were also impacted by the toxin. Collectively, these findings suggest that impairment of the major site of nutrient absorption in the small intestine may be part of the “collateral damage” that occurs after ETEC infection. While it is certainly premature to equate these changes with the development of EED and malnutrition, at the very least they suggest that we still have much to learn about the molecular pathogenesis of these pathogens that are virtually ubiquitous in regions lacking the basic human necessities of clean water and sanitation.

## FUTURE DIRECTIONS

Unfortunately, in the absence of a broadly protective vaccine, young children in low-middle-income countries will likely continue to bear the burden of acute diarrheal illness imposed on them by ETEC. Resolving the role of more recently discovered virulence proteins in the molecular pathogenesis of ETEC may focus vaccine development on antigens that encompass the breadth of ETEC.

While ETEC have been epidemiology linked to development of sequelae including malnutrition, additional study is needed at the molecular level to understand how these pathogens might promote changes in the small-intestinal architecture that underlie the sequelae. It is becoming clear, however, that one or both toxins secreted by ETEC can affect a variety of cellular pathways that govern normal homeostasis and ultimately absorptive capacity of the small intestine in addition to causing diarrhea. An ideal ETEC vaccine would mitigate or prevent both the acute diarrheal illness caused by ETEC, as well as the substantial burden of nondiarrheal morbidity linked to these pathogens.

## Notes

***Financial support.*** This work was supported by the National Institutes of Health (grants R01AI89894 and R01AI126887; Clinical and Translational Sciences award UL1 TR002345; and National Institute of Diabetes and Digestive and Kidney Diseases grant P30 DK052574 to the Washington University Digestive Diseases Research Core Center) and the US Department of Veterans Affairs (grant I01BX004825). Research reported in this publication was supported by the National Institute Of Allergy And Infectious Diseases of the National Institutes of Health under Award Number T32AI007172. The content is solely the responsibility of the authors and does not necessarily represent the official views of the National Institutes of Health.

***Potential conflicts of interest.*** J. M. F. is listed as inventor on US patent 8323668, “Prevention and treatment of gram-negative, flagellated bacterial infections,” which relates to the use of EtpA in vaccines. A. S. has no conflicts to declare. All authors have submitted the ICMJE Form for Disclosure of Potential Conflicts of Interest. Conflicts that the editors consider relevant to the content of the manuscript have been disclosed.
